# Provider and patient perspectives on barriers to buprenorphine adherence and the acceptability of video directly observed therapy to enhance adherence

**DOI:** 10.1186/s13722-019-0139-3

**Published:** 2019-03-13

**Authors:** Margo E. Godersky, Andrew J. Saxon, Joseph O. Merrill, Jeffrey H. Samet, Jane M. Simoni, Judith I. Tsui

**Affiliations:** 1 Division of General Internal Medicine, Department of Medicine, University of Washington, 325 9th Avenue, Seattle, WA 98104 USA; 2 Center of Excellence in Substance Abuse Treatment and Education, VA Puget Sound Health Care System, 1660 S Columbian Way, Seattle, WA 98108 USA; 3 Division of General Internal Medicine, Department of Medicine, Boston Medical Center, 801 Massachusetts Ave, Boston, MA 02118 USA; 4 Department of Psychiatry and Behavioral Sciences, University of Washington, 1959 NE Pacific St, Seattle, WA 98195 USA

**Keywords:** Qualitative research, Opioid use disorder, Opioid agonist therapy, Health technology, Adherence

## Abstract

**Background:**

Buprenorphine effectively reduces opioid craving and illicit opioid use. However, some patients may not take their medication as prescribed and thus experience suboptimal outcomes. The study aim was to qualitatively explore buprenorphine adherence and the acceptability of utilizing video directly observed therapy (VDOT) among patients and their providers in an office-based program.

**Methods:**

Clinical providers (physicians and staff; *n *= 9) as well as patients (*n *= 11) were recruited from an office-based opioid treatment program at an urban academic medical center in the northwestern United States. Using a semi-structured guide, interviewers conducted individual interviews and focus group discussions. Interviews were digitally recorded and transcribed verbatim. Transcripts were independently coded to identify key themes related to non-adherence and then jointly reviewed in an iterative fashion to develop a set of content codes.

**Results:**

Among providers and patients, perceived reasons for buprenorphine non-adherence generally fell into several thematic categories: social and structural factors that prevented patients from consistently accessing medications or taking them reliably (e.g., homelessness, transportation difficulties, chaotic lifestyles, and mental illness); refraining from taking medication in order to use illicit drugs or divert; and forgetting to take medication, especially in the setting of taking split-doses. Some participants perceived non-adherence to be less of a problem for buprenorphine than for other medications. VDOT was viewed as potentially enhancing patient accountability, leading to more trust from providers who are concerned about diversion. On the other hand, some participants expressed concern that VDOT would place undue burden on patients, which could have the opposite effect of eroding patient-provider trust. Others questioned the clinical indication.

**Conclusions:**

Findings suggest potential arenas for enhancing buprenorphine adherence, although structural barriers will likely be most challenging to ameliorate. Providers as well as patients indicated mixed attitudes toward VDOT, suggesting it would need to be thoughtfully implemented.

## Background

In the last decade, opioid use disorders (OUDs) have emerged as a public health crisis, with an estimated 2.4 million American adults living with an OUD [1]. In 2016, 42,249 people died from opioid-related overdoses in the United States [2]. OUDs are closely intertwined with injection drug use, hepatitis C (HCV), and HIV [3]. Expanding medication treatment for OUD and improving patient retention in treatment are critically needed to combat the epidemic and prevent adverse health outcomes, including early mortality.

Opioid agonist therapy, with either methadone or buprenorphine, has been shown to reduce illicit opioid use [4] and downstream complications such as overdose [5, 6], HIV [7], and HCV [8–10]. Buprenorphine, a partial µ-opioid receptor agonist, offers unique advantages over methadone, including less subjective euphoria and lower risk for side effects such as sedation [11]. Furthermore, buprenorphine can be prescribed by any trained/waivered provider in an office setting, as opposed to methadone, which must be dispensed in federally regulated opioid treatment programs. Thus buprenorphine delivery has the advantage of being scalable and patient centered [12]. Research on patient preferences has suggested that some patients choose buprenorphine over methadone for greater convenience and autonomy as well as less stigma [13, 14].

Unlike licensed methadone treatment programs, office-based buprenorphine treatment programs do not provide medications through directly observed therapy (DOT); instead, they rely on patients to independently take their medications as prescribed. This delivery model risks non-adherence to buprenorphine compromising patients’ treatment outcomes.

Medication adherence has been defined as the extent to which a person’s medication-taking behavior follows the recommendations of a health care provider [15]. Non-adherence is a common problem that undermines the potential benefits of new pharmacotherapies for a number of chronic diseases, including treatment for substance use disorders. It is estimated that adherence to long-term therapy for chronic illness in developed countries is as low as 50% [16, 17]. For certain diseases there is a wealth of literature on adherence and interventions to improve adherence. A recent systematic review of interventions for adherence found that the most frequently targeted conditions were HIV, depression, chronic obstructive pulmonary disease, cardiovascular disease, hypertension, and diabetes [18]. For HIV medications alone, a recent review of qualitative studies on adherence identified 127 studies [19], and such research has been critical to inform theoretical frameworks and the development of efficacious adherence interventions [20]. As with antiretroviral treatment for HIV (and many other chronic disease medications), the success of opioid agonist treatment for opioid use disorders is dependent on medication adherence, yet the topic has been relatively unexplored.

Buprenorphine has some unique aspects compared to medications for other chronic medical conditions. On the one hand, the potential for the development of physical tolerance and consequent withdrawal symptoms without use may be a strong deterrent for buprenorphine non-adherence among patients. On the other hand, there is a higher risk for diversion of this medication given the nature of its being a controlled substance, which may drive non-adherence. Diversion is the intentional or non-intentional transfer of medication to an unintended party through selling and/or trading, giving away, or loss of medication [21, 22]. In a sample of injection drug users with opioid use, illicit buprenorphine use was reported by 45% [23], and a recent study of persons entering inpatient treatment for medically supervised opioid withdrawal found that among those who had been prescribed buprenorphine, half admitted to “sharing” and a quarter reported selling medication [24]. Clearly, for some patients the desire to help other individuals or the temptation of financial gain through diversion may be a threat to adherence with their own medication.

Research suggests that buprenorphine non-adherence may be fairly common and associated with poorer outcomes. A study of patients treated with buprenorphine in an office-based setting that used computerized caps on medication bottles to monitor adherence reported that on average patients were adherent to their medication only 71% of the time [25]. Non-adherence to buprenorphine is associated with illicit drug use and non-retention [25–28]. Buprenorphine has a high affinity for opioid receptors and, when taken regularly, will alleviate opioid cravings and block the effects of other opioids, providing little incentive for illicit opioid use. However, buprenorphine’s effectiveness is dependent on adherence. Methadone is associated with better treatment retention rates compared to buprenorphine [29–31], perhaps in part due to delivery through a model of directly observed therapy.

Video directly observed therapy (VDOT) is a novel method of confirming medication ingestion using an asynchronous video platform via mobile phone application which is modeled after in-person directly observed therapy. With VDOT, patients are able to be observed in their own environment without the constraints necessary to attend a clinic for in-person observation or have clinical staff travel to patients. VDOT has been successfully utilized for the treatment of tuberculosis (TB) [32, 33] and was implemented post-disaster relief to provide medical monitoring for patients affected by Hurricane Katrina [34]. VDOT has been found to be acceptable among patients and providers for treatment of tuberculosis; however, it is unknown whether patients and providers would be receptive for its selective use with buprenorphine. While standard of care for treatment of OUD with methadone utilizes face-to-face DOT at federally approved opioid treatment programs, there is no such standard for monitoring adherence among patients who receive office-based buprenorphine. Yet, the delivery of office-based treatment with buprenorphine places a large burden for monitoring on providers and clinic staff [35, 36]. Currently there is a buprenorphine provider shortage, and known barriers to prescribing include fears of diversion [37]. Use of VDOT could potentially ameliorate providers’ concerns about diversion and decrease the need for frequent face-to-face visits and urine drug testing. Potentially, VDOT could preserve patient autonomy while allowing for sufficient monitoring to ensure safety and treatment success.

This study among patients and providers within a primary care office-based program aimed to examine perspectives on barriers to buprenorphine adherence, with specific focus on circumstances and reasons why patients might not take their medication regularly. A secondary intent of the study was to explore the acceptability of a mobile phone application for VDOT buprenorphine treatment for opioid use disorder.

## Methods

### Study design

We conducted a qualitative study of providers (physicians and staff) and patients receiving care at an office-based opioid treatment (OBOT) program through the use of individual, semi-structured interviews and focus group discussions comprising of 2 to 4 individuals each.

### Recruitment of study sample

Participants were recruited from an urban, safety net, academic medical center’s OBOT program embedded within an adult medicine primary care clinic between July and September 2017 in the northwest U.S. The OBOT program follows a collaborative care model whereby nurse care managers support physicians to provide care for patients [12]. The sample comprised two distinct groups: (1) providers (i.e. physicians, registered nurses, medical assistant, and the program manager) and (2) patients. All OBOT providers who were not part of the research team (i.e., authors JT and JM) were eligible to participate in interviews. Providers were individually contacted by research staff to assess interest in participation. Patients in the OBOT clinic were eligible if they were between the ages of 18 and 64 years, currently part of the OBOT program, and English speaking. Those showing signs of acute intoxication or cognitive impairment at the time of recruiting were excluded. Researchers were present during normal clinic hours to talk to interested patients referred by clinic providers. Patients were referred to a non-clinical study team member who was not previously known to patients. Patients were also provided with flyers including a study phone line if patients could not speak directly with researchers during their visit to the clinic. Providers were encouraged to refer all potentially eligible and interested patients. Patients were given a brief overview of the study objectives and requirements and given the opportunity to participate if deemed eligible. All potential patient participants were informed before and during the informed consent process that their participation in the study was voluntary and would not affect clinical services. We sought to enroll a total sample of all eligible providers. Patient recruitment was conducted until thematic saturation was felt to have occurred (i.e. new transcripts did not reveal new themes).

### Data collection

For efficiency, two focus groups for providers were scheduled, and those who could not attend either were scheduled for individual interviews. Patients were scheduled for individual interviews only. Initial interviews with providers were conducted by two members of the research team (JT and MG), while subsequent interviews were conducted with a single researcher (MG) who was not involved in the clinical care of patients. Interviews were conducted in a private, closed space and lasted between 15 and 45 min. Demographics were collected from all participants at the start of the interview. General, open-ended questions were used to collect information on experiences with buprenorphine non-adherence without imposing an expectation of responses. Providers/staff were asked, “What are some challenges to adherence to buprenorphine/naloxone that your patients face?” Probes for patient interviews were (1) “What are things that make it hard for patients to take their buprenorphine?” and (2) “Can you tell me about a time you stopped taking your medication or missed a dose?” To assess acceptability of VDOT, patients/providers were asked: “The researchers of this study are developing a smartphone-based app to allow patients to upload videos of themselves taking their medications daily (“video DOT”). What do you think about such a tool?” Interviews were professionally transcribed verbatim and reviewed by research staff for completion and clarity. Personal identifiers were censored. Written informed consent for participation, including audio recording and transcription of interviews, was obtained from all participants. All participants were compensated $20 via an electronic gift card for completion of the study interview. The Institutional Review Board at the University of Washington approved this study.

### Qualitative analysis

Two study team members (JT and MG) independently reviewed transcripts and identified thematically similar content that was coded to reflect conceptual categories with the intention of developing a thesis using grounded theory [38]. Transcripts were reviewed in chronologic order, and themes were continually expanded and refined over time. Nvivo 11 software [39] was used to capture themes. After the completion of coding for a few transcripts, the researchers met and reviewed their codebooks, themes and associated transcripts to develop consensus on coding schemes and main themes. This iterative process was repeated until all transcripts were reviewed by both researchers and the investigators had developed a series of themes that they felt reflected participants’ experiences and attitudes related to buprenorphine adherence and the use of VDOT.

## Results

### Sample characteristics

The final sample included 20 participants: 9 OBOT providers and 11 patients. All providers who were offered enrollment in the study agreed to participate. Seven participated in focus groups and two in individual interviews.

Providers’ mean age was 38.4 (± 8.9) years, and the majority were female (78%). Providers self-identified as White (67%), Asian (11%), multiracial (11%), or other (11%). Patients’ mean age was 36.2 (± 8.6) years; the majority were male (73%) and were White (81%). Others identified as Hawaiian/Pacific Islander (9%) and Latino (9%).

### Themes

#### Buprenorphine adherence

A number of themes emerged when discussing barriers to buprenorphine adherence. Notably, patients and providers pointed to *structural factors* which made consistent access to medications difficult. Providers pointed to patients’ *lack of transportation* as a barrier to attending appointments, a prerequisite for having medication. Providers also acknowledged this expectation of clinic attendance to receive medications as a potential challenge to medication adherence, setting up clinical dilemmas.

*Distance to clinic is a big* *[factor], like sometimes getting to visits, again that organization to be there at a certain time, because it’s labor intensive to come every week,* *[and have]* *transportation.* (Participant 11, provider)

*Patients have difficulty with transportation to appointments, and then they miss their appointment, and then they’re out of medication or we have to make a decision about sending medication when we haven’t seen them or don’t have any data about how their treatment is going. That’s a challenge for adherence*. (Participant 3, provider)

*Homelessness* was also identified as a major challenge for patients, both in the context of keeping appointments in order to have prescriptions filled and also taking medications regularly as instructed.

*Being homeless or not having something stable [so] you can feel comfortable, have a set schedule. It’s hard to have a set time to wake up in the morning if you don’t even have a bed to sleep in every night where you’re going to put your head down.* (Participant 4, patient)

*I think the challenges for some of my patients are that they are homeless or they have chaotic kind of life situations, and so that kind of becomes a priority sometimes above buprenorphine.* (Participant 10, provider)

Providers also noted *mental illness* as being a patient-level barrier to buprenorphine adherence for a small number of patients.

*We definitely have a couple people… who [are] very disorganized relating to mental health and paranoia. That affects his own belief about the medication and how he should take it and his motivation to take it.* (Participant 6, provider)

Another challenge that emerged was the persistent *desire to use illicit drugs*. Patients, in particular, were frank and open about their competing desires to take their buprenorphine that would block euphoric effects of using illicit opioids vs. skipping doses in order to use illicit drugs and get “high.”

*It’s just because us addicts, whether everyone else wants to admit it or not, we like to get high. And taking the medicine, we can’t get high if we’re taking it like we should…That’s why I wouldn’t want to take it all the time.* (Participant 17, patient)

Providers noted that this temptation to skip doses in order to use was particularly strong for patients who were recent initiators of buprenorphine. Therefore, this particular threat to buprenorphine adherence was more common among patients early in their treatment.

*[Skipping doses typically occurs]* *pretty early on in treatment where they have the misconception that once they have taken enough doses to have enough buprenorphine saturating their receptors, that if they use heroin it’s going to precipitate withdrawal. … Sometimes people will intentionally skip in order to use. Or what is more frequent is a person will use and then skip their next couple of doses, kind of start all over from the beginning.* (Participant 3, provider)

Some providers and patients mentioned *forgetting medication* as a reason for non-adherence, especially in the setting of taking split-doses.

*Sometimes I have [forgotten to take my buprenorphine], in the afternoon, when you’re busy and not really thinking about it …There has been times that I have just [said] throughout the day, “Hey, I forgot to take my Suboxone (sic).”* (Participant 14, patient)

However, the distinctions between “forgetting” and other motivations, such as refraining from taking buprenorphine to use drugs, were not always clear.

*[A barrier is] just like forgetfulness, get busy doing things, or definitely if I’m screwing up and getting intoxicated or drinking or whatever. Like when my friend just came to town. So that’s just always what happens when we’re together. … [We get] busy talking all day, catching up with everything. I haven’t seen him for a long time. So then I just be like, “Did I take my evening dose?”* (Participant 19, patient)

Patient and provider participants also brought up the issue of *diversion* as a possible reason for non-adherence, and one that is particularly difficult to measure.

*I think that sometimes we don’t know if all the medication we’ve prescribed to a specific person is going to that specific person. We wonder if some of it is going to somebody else.* (Participant 3, provider)

*I also can’t be entirely confident that people are taking the entire does of the medicine. Of course we’re monitoring that they’re taking it [with urine drug tests]. So is it possible that they’re not taking all of it? We know that there’s diversion. … I guess diversion could in a way be kind of an adherence challenge.* (Participant 6, provider)

*I think the current system seems to work, although I do know a lot of people who sell their Suboxone.* (Participant 9, patient)

However, providers also acknowledged the social pressures that patients might face that could lead to diversion and the reluctance to discuss diversion with them because of fear of consequences; patients may have been similarly inhibited to bring this up in interviews and none reported diversion as a form of their own non-adherence.

*I think a lot of our patients are in communities of people who are in various stages of use or recovery. And also communities that have a strong ethic of mutual support. They want to help each other. And what we consider diversion they consider helping their friends or their spouses or their family…[so]challenges like “It’s hard for me to have bus fare,” we hear more about rather than, “Oh, I needed to give half my medication to my friend.” We never hear about that because patients are aware that the consequence for that is very serious.* (Participant 3, provider)

Not all participants endorsed that there were challenges to buprenorphine adherence: there were both *providers and patients who did not perceive difficulties*.

*I can only speak for myself and I don’t feel I have an issue with it. I like taking my meds; I look forward to taking the medication every day.* (Participant 13, patient)

*There are certainly patients that don’t take the medication, but I feel like that’s less common with buprenorphine than some other medications that I prescribe.* (Participant 7, provider)

Some participants noted that there was already a *strong incentive to adhere* to buprenorphine given that missing doses could result in withdrawal symptoms.

*I have many patients that tell me, ‘I wake up in the morning, and I start to feel just a little anxious or irritable, that I could be having withdrawal symptoms soon, and that’s my trigger to say I need to take my dose.’* (Participant 7, provider)

However, those same withdrawal symptoms could also be a trigger for illicit drug use that could undermine adherence and recovery.

*Well, know that if you don’t take it on time and stuff, you might get withdrawals. And so that’s always in your mind, too. You want to always make sure that you’re not feeling that kind of withdrawal or a bad feeling because that might make you want to go out and use.* (Participant 15, patient)

#### VDOT acceptability and utility

Both providers and patients generally responded favorably to the idea of VDOT for buprenorphine and perceived the utility of VDOT in addressing some of the barriers described above. They acknowledged it might help patients to remember to take their medication and *might facilitate a stronger connection with providers*. The use of VDOT was viewed as a way for patients to actively participate in their recovery and *show accountability* to the program. Participants saw this as a means to increase trust between providers and patients

*Yeah, I think overall it would be a positive thing, especially when you’re first starting out and stuff. It would just help you take your meds on time and be connected to your providers and stuff. So, I’d be excited about it.* (Participant 15, patient)

*I think a lot of it, the Suboxone program in general relies on a lot of trust and communication between the patient and provider, or providers, you know… So I think that [VDOT] would be good. Everybody could be on the same page. They feel good about it, especially when you’re changing doses or have had maybe problems in the past staying on the program. I think it would help hold people accountable*. (Participant 13, patient)

Providers also perceived that it might be particularly *helpful for patients who were newly engaged* in OBOT and might be struggling to adhere. They also brought to attention that it could be helpful to evaluate their patient’s technique, recalling patients who had in the past incorrectly taken their medication.

*I think sometimes our patients just need more, especially in the beginning phases of coming on board with medications as a treatment, probably just need a bit more monitoring. And/or making sure they’re taking their medication properly. And/or taking the medication at all… [VDOT is] a good way for out providers to ensure that the medication is going to the right people.* (Participant 1, provider)

*Sometimes people say “I don’t feel like I’m getting the full effect of the medication, it takes forever to dissolve.” And I’ll say, “Let’s do an observed dose. I want to see how you’re taking medication in case there are suggestions I could offer you that would be helpful.” Sometimes I’ve found patients doing really strange stuff with their medication. One person was crushing it and drinking it…* “ (Participant 3, provider)

*Where I think it [VDOT] would be beneficial, for example, I had a patient the other day who said, “Those pills just weren’t working for me. I was having trouble getting under my tongue and they were sticking to my fingers.” And if we had a video of that, we could sit and look at those and say, “Let’s watch this and see if we can identify where you’re having difficulty.”* (Participant 18, provider)

VDOT was also perceived by providers as a *useful tool to safeguard against diversion*.

*I remember we had a new patient. This person came from in*-*patient. Had a lot of steps, a lot of appointments in different places. Got opiates, [then] got buprenorphine from us…. And then got prescribed opiates. There was a big question mark, I think, like what medication is this person taking? How do we know? Do we keep treating this person? Are they diverting the medications? [It would be helpful] in that case seeing if they’re taking the medications through video.* (Participant 2, provider)

Patients also felt that VDOT could be *a way to overcome logistical issues* of not having transportation or enough time to adhere to appointments to receive medication.

*Sometimes you can’t make it all the way to the doctor’s office, or you have to work and you can’t always make it there. But just being able to know that you can connect with a professional basically have your appointment taken care of, but you’re not really there, you know? It would open up a lot of time where people could have a more personal relationship with their doctor and provider. I think that could help in so many ways.* (Participant 4, patient)

However, a patient also pointed to *logistical barriers* that would exist to accomplishing VDOT itself.

*I personally wouldn’t have time to do that [use VDOT], though. I’m a nurse so I take mine when I get into work in the morning and I’m not allowed to have my phone on me at that point.* (Participant 20, patient)

A few participants and providers *questioned the need for VDOT*, even though they acknowledged barriers to adherence. Participants wondered whether treatment of OUD with buprenorphine warranted a different standard of care compared to other medication regimens.

*I mean, to me, it’s just taking medication…I guess I don’t really see a huge benefit in the whole thing because it’s like would you*– *there’s a lot of people that are on medications… I’ve seen people that have…to take like 20 a day. Would it be beneficial for them? I don’t think so either. So I just don’t know. I’m trying to think of the relevance of actually taking the medication and watching yourself do it.* (Participant 5, patient)

*There are certainly patients that don’t take the medication, but I feel like that’s less common with buprenorphine than some other medications I prescribe.* (Participant 7, provider)

Though many participants saw potential benefits of VDOT in the OBOT program, there were concerns about the adverse effects of increased monitoring, that it could *erode patient trust*.

*It’s yet another obligation on the patient’s time and expectation that they have to meet that may feel like is indicative of a lack of trust on the part of the provider.* (Participant 8, provider)

One patient had a strong negative response to the idea of VDOT for buprenorphine, fearing that it would *compromise privacy*.

*The privacy, the invasion of privacy is just way too huge on that…There is no way they can guarantee that that is going to…stay secure and stay private.* (Participant 12, patient)

Additionally, there were *practical concerns* that patients would be unable to learn to use the technology and that limited access to smart phones and wifi would be a barrier to adoption. Providers also voiced concern that among this patient population smartphones would be at risk for getting stolen. In general, there appeared to be cautious optimism that this technology could be useful for some, but not all, patients.

*I know that there’s some people that just aren’t technologically savvy, you know? They don’t know how to use the phones and stuff. And there’s people that it might be like*– *it might make them frustrated and they just might not want to mess with it if they can’t figure it out.* (Participant 15, patient)

*A lot of patients have phones that aren’t smart phones and get lost or they don’t have money to pay for the service*. (Participant 6, provider)

*I think it’s a great idea, the app, to make sure they’re taking it and how they’re taking it. My biggest concern is my patients get everything stolen*–*even if they had just a glucometer or things that aren’t valuable people get them stolen. I would think that a smartphone would get stolen pretty regularly.* (Participant 10, provider)

*I know a lot of my patients do have phones that have very limited data plans, if any, or they are just minutes that run out pretty readily. So they are not in possession of devices they can just kind use these types of apps. And then there are patients [who] I feel their technological literacy is such that it is something they’d be really engaged with.* (Participant 18, provider)

*I think there’s definitely a benefit. I think patients are always willing to try something new. I have been impressed over the last decade as technology has evolved… So, I think it’s always worth a try.* (Participant 18, provider)

## Discussion

Through qualitative interviews with providers and current patients at an office-based buprenorphine program based in an adult primary care clinic in the northwest U.S., we identified reasons for buprenorphine non-adherence. Among both groups, perceived reasons for buprenorphine non-adherence generally fell into two categories: factors that prevented patients from being able to consistently access their medications (e.g. homelessness, transportation difficulties, and chaotic lifestyles) and those that resulted in patients not taking their medications as instructed even when medications were available. Reasons for the latter category included intentionally withholding medication in order to use illicit drugs, diversion of medication and forgetting to take medication. Generally, providers and patients alike were receptive to the idea of using VDOT for buprenorphine treatment. Perceived benefits were enhanced patient accountability, leading to more trust from providers who are concerned about diversion. On the other hand, patients and providers expressed some concerns that it would place undue burden on their patients, and some questioned the need for such an adherence monitoring for buprenorphine.

This study sheds new light on the phenomenon of non-adherence to buprenorphine treatment for patients with opioid use disorders. Buprenorphine has proven efficacy; however, in the real world, competing factors may interfere with patients’ ability to be fully adherent to medication, which can jeopardize important treatment outcomes such as retention. Although there are a number of studies that have examined predictors of buprenorphine treatment initiation and retention [40–46], we are unaware of other studies that have focused on medication adherence. A prior qualitative study also explored facilitators to retention in buprenorphine treatment and found positive reaction to medication, personal commitment, and support from clinic staff to be facilitators, whereas transportation issues and competing priorities were barriers [47]. Our study also identified homelessness and transportation to be barriers to adherence, pointing to a critical need for social services, as well a possible role for mobile health technologies which might cut back on the need for frequent face-to-face visits for patients with transportation difficulties. Patients did mention occasional forgetting to take medication, although in general this was mitigated by withdrawal symptoms. However, interviews revealed that the daily decision whether to take their medication or to use drugs was a challenge. Such challenges to adherence might be addressed by switching to use of extended release formulations of buprenorphine [48], as well as use of mobile technology for VDOT, text messaging and other supportive features [49–51].

Diversion was acknowledged as a potential reason for medication non-adherence among patient and provider participants interviewed in this study. Providers, but not patients, identified VODT as a potential tool to monitor for diversion. This suggests differing perspectives on the usefulness of VDOT as a tool for adherence monitoring versus diversion control, and the distinctions therein. While diversion requires non-adherence, adherence monitoring is not the same as diversion control. Reports of misuse and diversion have emerged in the U.S. over time and have prompted recommendations for the development of diversion policies for office-based buprenorphine programs [11, 52–54]. A study of buprenorphine providers found that at least half felt that diversion was at least a moderately significant problem in their communities, and another observed that among rural providers, diversion was cited as the leading barrier (48%) to prescribing buprenorphine [35, 37]. Research shows that providers do employ various strategies to reduce diversion [35, 36]. However, many of those strategies are not convenient to patients, such as requiring more frequent than monthly visits and regular urine drug testing, and some may even be counter-productive to engaging and retaining patients, such as limiting doses, selectively accepting patients and immediately terminating patients with suspected diversion. Though VDOT would not measure diversion directly, measuring adherence may serve to alleviate some of the unknown felt by providers. There is a need for additional diversion control strategies such as VDOT which might both mitigate provider fears and allow more flexibility for patients. The use of VDOT would not be a substitution for but rather an addition to existing clinic approaches for reducing diversion. Shortages of buprenorphine prescribers exist in many areas of the country in part because of providers’ concerns about diversion [37, 55–57]; having additional tools to safeguard against diversion might increase providers’ willingness to prescribe.

Based on our qualitative interviews, it appeared VDOT for buprenorphine was acceptable for most providers and patients. This is the first study of which we are aware to solicit patient and provider views on the acceptability of VDOT for buprenorphine treatment. A previous qualitative study examined patient and provider perspectives on use of VDOT for monitoring TB treatment in the U.S.-Mexico border region and found broad support for use of the technology in both groups, as did a pilot study of its use in TB public health clinics in Maryland [58, 59]. As with our study, providers also expressed concern for patient literacy and technical support to allow them to successfully use this technology. In contrast with those study, our study did reveal some opinions that VDOT might be unnecessary for this treatment as there are other compelling reasons to adhere (such as avoiding withdrawal symptoms), and DOT is not currently the standard of care for buprenorphine treatment. Yet some patients found the structure and greater accountability from VDOT appealing, which prior studies have suggested patients desire from treatment [14]. Indeed, the requirement for daily observed dispensing may be one factor that contributes to better retention rates among methadone treated patients compared to buprenorphine [60]. It is possible that retention to buprenorphine treatment could improve with the addition of VDOT among primary care office-based programs; however more research is needed to prove such benefits. While forgetting to take medication was mentioned as one reason for non-adherence, it is unclear if patients would be less likely to “forget” with prompts for VDOT. Furthermore non-adherence to VDOT (due to “forgetting” or inconvenience) could exceed that for medication non-adherence, limiting its utility. Finally, it should be noted that among the patients interviewed, there was one individual who was of the strong opinion that he would never utilize such technology due to the fear that it would put data security and privacy at risk. As such, it is clear that not all patients will be willing to partake in such an adjunct to buprenorphine treatment.

This study had a number of limitations. Participants were recruited from a single site in an urban hospital-based clinic that adopted a collaborative care model utilizing nurse care managers based on prior experience in Massachusetts [12]. This sample may differ from other office-based buprenorphine settings. Our study sample size was relatively modest, and for patients was limited to a convenience sample. We did not collect information on length of treatment from patient participants. It is possible that perspectives on VDOT might differ among patients who are more newly engaged. We used relatively few prompts to solicit information on barriers to buprenorphine from patients and providers in order not to impose a pre-existing framework given the exploratory nature of this work, but this may have limited the number of themes that emerged. There is the possibility that social desirability bias may have influenced participants’ responses; we attempted to mitigate this by having a researcher (MG) uninvolved with clinical care conduct the bulk of the interviews.

## Conclusion

In summary, this study demonstrates that there are a number of scenarios in which patients do not adhere to taking buprenorphine, including a desire to refrain from taking medication in order to use illicit drugs and also simply forgetting. As such, it appears that interventions to improve adherence may be of benefit to patients with opioid use disorders who are treated with buprenorphine. This study also suggests that patients and providers are receptive to the idea of using mobile health technology to allow VDOT of buprenorphine treatment. Further research is needed to test whether VDOT is effective for improving clinical outcomes among patients who are treated with buprenorphine for opioid use disorders, and whether it has utility as a diversion control strategy for providers.

## Abbreviations

DOT: Directly observed therapy
VDOT: Video directly observed therapy
OBOT: Office-based opioid treatment
OUD: Opioid user disorder
HCV: Hepatitis C virus
HIV: Human immunodeficiency virus
TB: Tuberculosis
